# Association Between ASA Physical Status Classification and Postoperative Intensive Care Requirement in Pediatric Patients Undergoing Dental Treatment Under General Anesthesia: A Retrospective Study

**DOI:** 10.3390/healthcare14050615

**Published:** 2026-02-28

**Authors:** Enes Bardakci, Mehmet Sinan Dogan, Peris Celikel

**Affiliations:** 1 Department of Pediatric Dentistry, Faculty of Dentistry, Harran University, Sanliurfa 63050, Turkey; enesbardakc67@harran.edu.tr (E.B.); drmsdogan@harran.edu.tr (M.S.D.); 2 Department of Pediatric Dentistry, Faculty of Dentistry, Ataturk University, Erzurum 25240, Turkey

**Keywords:** ASA physical status classification, pediatric dentistry, general anesthesia, systemic diseases, postoperative intensive care, healthcare resource planning

## Abstract

**Background and aim**: Accurate perioperative risk stratification is essential for patient safety in pediatric dental treatment under general anesthesia. We aim to evaluate the association between ASA (American Society of Anesthesiologists) Physical Status Classification and postoperative intensive care requirement in pediatric patients undergoing dental treatment under general anesthesia. **Methods**: In this retrospective study, the clinical records of 1003 children who underwent dental treatment under general anesthesia between June 2022 and June 2025 were evaluated. The patients’ age, gender, ASA classification, concomitant systemic diseases, and postoperative intensive care requirements were analyzed. The chi-square test and logistic regression analysis were used for statistical evaluation, and results are expressed as odds ratios (ORs) with 95% confidence intervals (CIs). **Results**: The mean age of the patients was 5.78 ± 2.87 years, and 58.3% were male. All patients requiring postoperative intensive care were in the ASA II (34.7%) and ASA III (65.3%) groups, while no intensive care need was observed in the ASA I group (*p* < 0.001). The need for intensive care significantly increased, particularly in the presence of cerebral palsy, epilepsy, autism, congenital heart disease, and intellectual disability (*p* < 0.001). In addition, the mean age of children requiring intensive care was significantly higher (*p* < 0.001). In multivariable logistic regression analysis, ASA classification was significantly associated with postoperative intensive care requirement (OR = 180.73, 95% CI: 9.40–1922.49, *p* < 0.001), whereas age and gender were not independently associated. Furthermore, the interaction term between ASA and age (ASA × Age) was not statistically significant (*p* = 0.59). **Conclusions**: ASA classification was significantly associated with postoperative intensive care unit admission in pediatric patients undergoing dental treatment under general anesthesia and may contribute to perioperative risk assessment. The findings emphasize the need for early identification of high-risk children and support the integration of ASA classification into multidisciplinary preoperative planning to enhance patient safety and optimize postoperative resource utilization.

## 1. Introduction

Dental caries is one of the most common chronic diseases worldwide, with untreated dental caries affecting approximately 0.62 billion children [1,2]. Recent global health reports continue to identify early childhood caries as a major public health concern, particularly in low- and middle-income countries, where access to preventive care remains limited. Delays in dental treatment in the pediatric population may lead to pain, infection, nutritional and sleep disorders, school absenteeism, and limitations in daily activities, thereby negatively affecting children’s physical and psychosocial well-being. The main reasons for these delays include dental anxiety, fear, and lack of cooperation commonly seen in children, which highlight the limitations of traditional clinical approaches in pediatric dentistry [3].

In this patient group, advanced behavior management strategies are essential to ensure effective and safe dental treatment. Due to dental anxiety in pediatric patients, sedation and general anesthesia (GA) are frequently used in pediatric dentistry, along with non-pharmacological behavior guidance methods [4,5]. The American Academy of Pediatric Dentistry (AAPD) states that dental care is medically necessary to prevent and eliminate orofacial diseases, pain, and infection; restore the form and functionality of teeth; and correct facial deformities or dysfunction [6].

In pediatric patients in whom cooperation cannot be achieved, comprehensive dental care performed under general anesthesia has been reported to be more effective and more cost-effective than repeated sedation procedures [7,8,9]. Furthermore, previous studies have reported increased restoration longevity following treatment under general anesthesia. Early treatment under general anesthesia has also been associated with improved cooperation in subsequent dental visits. With the increasing utilization of GA in pediatric dentistry, accurate perioperative risk stratification has become increasingly important for both patient safety and healthcare resource allocation [10,11,12].

To ensure perioperative patient safety in children scheduled for dental treatment under general anesthesia, accurate assessment of their systemic health status is critical. One of the most commonly used tools for this purpose is the ASA (American Society of Anesthesiologists) Physical Status Classification developed by the American Society of Anesthesiologists. Although initially developed for adult patients, the ASA classification is now widely used in the pediatric population. In contemporary clinical practice, the ASA classification is frequently used not only as a descriptive system but also to support perioperative clinical assessment [6,13].

Due to the diversity of childhood diseases and their systemic effects, the reliability of the ASA classification in pediatric patients and inter-rater agreement remain controversial [13,14]. A multicenter study by Ferrari et al. [13] reported limited inter-rater agreement regarding the ASA classification in pediatric patients. However, the association between ASA classification and postoperative intensive care requirement in pediatric dental anesthesia settings has not been sufficiently explored. Particularly in dental settings, where procedures are often elective yet involve vulnerable populations, the prognostic implications of ASA scoring warrant further investigation.

Recent studies have shown that children with high ASA scores have more anesthesia-related complications and a greater need for postoperative intensive care [14,15]. Systemic diseases such as epilepsy, autism spectrum disorder, congenital heart disease, cerebral palsy, and Down syndrome directly affect the ASA score and perioperative risk level [16].

The importance of ASA classification in the dental field is becoming increasingly evident in pediatric dentistry practices. The presence of systemic diseases in pediatric patients scheduled for dental treatment under general anesthesia not only increases the risk of anesthesia but may also necessitate postoperative intensive care. In this context, when planning treatment in pediatric dentistry, it is crucial to evaluate the relationship between the ASA classification and the burden of systemic disease and the potential need for intensive care. A clearer understanding of this relationship may contribute to improved multidisciplinary planning, optimized allocation of intensive care resources, and enhanced patient safety [17]. The aim of this study was to retrospectively evaluate the association between ASA Physical Status Classification and postoperative intensive care requirement in pediatric patients undergoing dental treatment under general anesthesia. The null hypothesis (H_0_) was that there is no significant association between ASA Physical Status Classification and postoperative intensive care requirement in pediatric patients undergoing dental treatment under general anesthesia.

## 2. Materials and Methods

### 2.1. Study Design and Ethical Approval

This retrospective observational analytical study was conducted by reviewing the clinical records of pediatric patients who underwent dental treatment under general anesthesia at the Department of Pediatric Dentistry, Faculty of Dentistry, Harran University, between 2022 and 2025. All data were anonymized, and patient identification information was not used in the analysis process. The study protocol was approved retrospectively by the Harran University Clinical Research Ethics Committee due to the retrospective nature of the study (Decision No: HRU/25.19.63; Date: 12 January 2025), after completion of data collection. No additional data were collected after ethical approval was granted.

### 2.2. Study Group and Inclusion Criteria

The inclusion criteria for the study were defined as being between 2 and 18 years of age, having undergone dental treatment under general anesthesia, and having complete data in the patient file regarding ASA classification, systemic disease information, and postoperative intensive care needs. Patient records with missing, incorrect, or insufficient data were excluded from the study. Patients classified as ASA IV or higher were excluded due to the small sample size and the distinct perioperative management protocols applied to this high-risk group.

### 2.3. Data Collection and Variables

Data obtained from patient records included age, gender, ASA Physical Status Classification (ASA I, ASA II, ASA III), presence and type of systemic diseases (epilepsy, cerebral palsy, autism spectrum disorder, congenital heart disease, etc.), type of dental treatment performed, duration of anesthesia, and need for postoperative intensive care.

Postoperative intensive care requirement was defined as admission to a dedicated pediatric intensive care unit (ICU), distinct from routine post-anesthesia care unit (PACU) monitoring. In our institution, postoperative recovery was initially assessed using the Modified Aldrete Score [18]. Patients with a total Modified Aldrete score < 8 or those exhibiting clinically significant respiratory, hemodynamic, or neurological instability were considered for ICU transfer.

Documented clinical indications for ICU admission included the need for invasive or non-invasive ventilatory support, persistent oxygen desaturation (SpO_2_ < 92% despite supplemental oxygen), hemodynamic instability requiring vasoactive support, continuous hemodynamic monitoring beyond standard PACU observation, or significant neurological impairment requiring close observation. Routine PACU monitoring without these criteria was not classified as an ICU requirement.

ICU admission was not routinely planned preoperatively but was determined postoperatively based on structured clinical assessment. Although Modified Aldrete scoring and predefined clinical parameters guided decision-making, final ICU transfer decisions were made by the attending anesthesiologist according to the patient’s overall clinical status and institutional practice.

### 2.4. ASA Physical Status Classification

Patients’ systemic health status was classified by anesthesiologists according to the current guidelines of the American Society of Anesthesiologists [19], based on preoperative assessment forms; ASA I includes healthy individuals without systemic disease; ASA II includes individuals with mild systemic disease; and ASA III includes individuals with serious but controlled systemic disease.

ASA classifications were performed by anesthesiologists working at the institution with pediatric anesthesia experience, following a standard preoperative assessment protocol.

### 2.5. Statistical Analysis

Statistical analyses were performed using IBM SPSS Statistics 25.0 (IBM Corp., Armonk, NY, USA) software. In descriptive statistics, categorical data are presented as numbers and percentages (%), while continuous data are presented as mean ± standard deviation and median (minimum–maximum). Due to the non-normal distribution of continuous data, the Mann–Whitney U test was used for comparisons between the two groups. The Kruskal–Wallis test was additionally used to compare age distributions across ASA classification groups. Relationships between categorical variables were evaluated using the Chi-Square test.

To determine the independent variables affecting postoperative intensive care requirement, penalized likelihood logistic regression using Firth’s correction was applied due to the absence of ICU events in the ASA I group and the resulting risk of quasi-complete separation in conventional logistic regression. Intensive care need (present/absent) was considered as the dependent variable; ASA classification (entered as a categorical variable with ASA I as the reference category), age, and gender were included as independent variables. An interaction term (ASA × age) was introduced to evaluate potential effect modification by age. The presence of systemic disease was not included in the final model due to conceptual and statistical collinearity with ASA classification. Results are reported as odds ratios (ORs) with 95% confidence intervals (CIs). The analysis was conducted using the logistf package in R (version 4.5.2; R Foundation for Statistical Computing, Vienna, Austria). Statistical significance was set at *p* < 0.05.

## 3. Results

### 3.1. Study Population

A total of 1003 pediatric patients who underwent dental treatment under general anesthesia at Harran University Faculty of Dentistry between 2022 and 2025 were included in the study. Of the patients, 585 (58.3%) were male and 418 (41.7%) were female. The findings were analyzed in terms of gender, ASA classification, presence of systemic disease, and postoperative intensive care needs.

### 3.2. Clinical Characteristics by Gender

Table 1 shows the distribution of intensive care needs, ASA classification, and accompanying systemic diseases in pediatric patients by gender. The rate of intensive care need was higher in male children than in female children, but this difference was not statistically significant (*p* = 0.068). No significant difference was found between genders in terms of ASA classification (*p* = 0.325). In contrast, the distribution of systemic diseases showed a significant difference according to gender (*p* < 0.001). Autism, cerebral palsy, epilepsy, and Down syndrome were more common in male children, while intellectual disability was detected at a higher rate in female children.

### 3.3. Age and Clinical Status

Table 2 shows the comparison of age means according to the children’s gender. The age mean of boys was determined as 5.83 ± 2.98 years, while the age mean of females was determined as 5.72 ± 2.73 years. The Mann–Whitney U test revealed no statistically significant difference in the mean ages according to gender (*p* = 0.586).

Table 3 presents a comparison of the mean ages of children according to their postoperative intensive care needs. The mean age of children requiring intensive care was 8.08 ± 4.32 years, while the mean age of children not requiring intensive care was 5.24 ± 2.07 years. The Mann–Whitney U test revealed that the mean age of children requiring intensive care was significantly higher (*p* < 0.001).

### 3.4. ASA Classification and Postoperative Intensive Care Requirement

Table 4 shows the relationship between postoperative intensive care need in children and ASA classification and accompanying systemic diseases. The vast majority of patients requiring intensive care were in the ASA III (65.3%) and ASA II (34.7%) classes, while no intensive care need was detected in the ASA I group. This distribution was found to be statistically significant (*p* < 0.001).

The results of the multivariable Firth penalized logistic regression analysis are presented in Table 5. Higher ASA classification was independently associated with an increased likelihood of postoperative intensive care requirement (OR = 180.73, 95% CI: 9.40–1922.49, *p* < 0.001). Age and gender were not significantly associated with postoperative ICU requirement in the adjusted model (*p* > 0.05). Furthermore, the interaction term between ASA classification and age (ASA × Age) was not statistically significant (*p* = 0.89), indicating that the effect of ASA classification on ICU need did not vary significantly according to age.

## 4. Discussion

The marked increase in intensive care requirement across ASA classes (ASA I: 0%, ASA II: 34.7%, ASA III: 65.3%) suggests a perioperative risk gradient consistent with increasing systemic disease burden. As the ASA classification reflects preoperative systemic health status by definition, this distribution is consistent with the structured risk hierarchy inherent to the ASA framework. In this context, the present findings demonstrate a significant association between ASA classification and postoperative intensive care requirement in pediatric patients undergoing dental treatment under general anesthesia.

In this study, none of the patients classified as ASA I required postoperative ICU admission. This observation is consistent with the lower systemic risk profile characteristic of this group. Nevertheless, institutional postoperative management practices, where clinically stable low-risk patients were routinely monitored in the PACU and discharged without planned ICU admission, may have influenced this finding. Moreover, as outcomes were assessed only within the first 24 postoperative hours, delayed adverse events could not be evaluated.

In contrast, the finding that more than two-thirds of children in the ASA III group required postoperative intensive care indicates that ASA classification may be associated with greater postoperative monitoring needs. The null hypothesis was therefore rejected, demonstrating a statistically significant association between ASA classification and postoperative intensive care requirement. These findings indicate that ASA classification, as a preoperative summary of systemic health status, was associated with postoperative intensive care requirement in this study. However, given the retrospective and single-center design of this study, these findings should be interpreted cautiously and cannot establish causality. The OR observed for ASA classification was accompanied by a wide confidence interval, reflecting the sparse data structure and the absence of ICU events in the ASA I group. Although penalized logistic regression was applied to obtain more stable estimates, the magnitude of the effect should still be interpreted with appropriate caution due to the extreme distribution of events across ASA categories.

Compared to the existing literature, these findings are largely consistent with previous reports; however, this study contextualizes the ASA–ICU association within a pediatric dental general anesthesia setting, a clinical field in which such data remain relatively limited. Hieronymus et al. [20] reported that 51.7% of children with special needs were in the ASA III class and that dental treatments in the vast majority of this group were performed under general anesthesia. This finding is consistent with this study, which found postoperative intensive care requirements in 65.3% of children in the ASA III group, supporting the notion that a high ASA class is an important factor associated with not only treatment requirements but also the level of post-treatment care. Similarly, Glessner et al. [21] reported higher rates of periodontal disease and tooth loss in individuals with ASA III classification, emphasizing the impact of increased systemic disease burden on oral health. This finding parallels the placement of children with high systemic disease burden in higher ASA classes in our study. Considering that neurological and developmental conditions such as epilepsy, cerebral palsy, and autism elevate the ASA score, it is clear that the general health status is closely related to pediatric dental treatment planning and postoperative follow-up requirements

It has been reported that developmental dental anomalies are more frequently observed in children with ASA II or higher, and that this condition is significantly associated with systemic diseases [22]. Similarly, our study also showed that neurological and developmental disorders were significantly more prevalent in children in the ASA II and ASA III groups. This finding suggests that the ASA score may indirectly reflect not only anesthesia risk but also dental and neurodevelopmental risks.

There are also studies that highlight the subjective aspects of the ASA classification. Tollinche et al. [23] reported serious interobserver discrepancies in the ASA classification of pediatric oncology patients. Similarly, Kwa et al. [24] showed that inconsistencies in ASA classification between anesthesiologists and surgeons increased the 30-day mortality risk.

In contrast, the fact that ASA scoring in this study was performed by anesthesiology specialists in accordance with standard guidelines important. Porcaro et al. [25] reported that the length of hospital stay was prolonged in individuals classified as ASA III, independent of age and body mass index. In this study, the significantly higher mean age of children requiring intensive care suggests that the burden of systemic disease and the associated anesthesia risk increase with age. This situation supports the need for more careful preoperative evaluation and postoperative monitoring in pediatric patients in the older age group.

Muslu Dinc ve Arun [26] reported that children in the ASA I group underwent more day-case surgical procedures, while in the ASA II and III groups, the duration of anesthesia and surgery was longer. Similarly, in our study, the significant increase in both the systemic disease burden and the need for intensive care in children in the ASA III group highlights the clinical value of the ASA score in terms of postoperative patient management and resource planning. Finally, Choi et al. [16] reported that dental treatments under general anesthesia are safer and more effective in individuals with severe disabilities. This finding further supports the need for close monitoring of children with higher ASA classes both during treatment and in the postoperative period.

In this study, the significantly higher mean age of children requiring intensive care suggests that the burden of systemic disease and the associated anesthesia risk increase with age. The higher ICU requirement observed among older children may be clinically explained by several factors. Older pediatric patients in this cohort were more likely to present with established or more complex systemic conditions, which may increase perioperative monitoring needs. Additionally, longer or more extensive dental procedures under general anesthesia in older children may contribute to prolonged anesthesia exposure and increased postoperative surveillance. These factors may collectively explain the observed association between age and ICU requirement.

The findings of this study should be evaluated within certain limitations. Due to the retrospective design, the analyses relied exclusively on existing medical records, which may have contained incomplete, inconsistently documented, or inaccurate information. The potential incompleteness of clinical files and variability in documentation practices may have affected data accuracy. Systemic disease information and ASA classifications were extracted from preoperative assessment forms; although ASA scoring was performed by experienced anesthesiologists following institutional protocols, the inherently subjective nature of the ASA classification and possible interobserver variability cannot be entirely excluded. Furthermore, undocumented comorbidities or variations in clinical judgment may have influenced risk categorization. Postoperative outcomes were assessed only within the first 24 h following general anesthesia, focusing specifically on the requirement for intensive care during this early period. Longer-term postoperative complications, delayed adverse events, or extended clinical outcomes were not evaluated. While ICU admission decisions were guided by predefined institutional criteria, the final transfer decision involved clinician judgment, which may introduce some degree of variability in postoperative outcome classification.

In addition, systemic diseases were evaluated based solely on their documented presence, and detailed information regarding disease severity, clinical staging, or level of control (degree of compensation) was not consistently available. Variations in the control status of systemic conditions may have influenced perioperative risk assessment, ASA classification, and postoperative intensive care requirements. Therefore, the absence of standardized severity grading limits a more nuanced interpretation of the relationship between systemic disease burden and clinical outcomes.

Nevertheless, the study’s large sample size, focus on the pediatric dental population, and reliance on real clinical data are strengths that highlight the clinical and prognostic importance of ASA classification in pediatric dentistry. Future prospective, multicenter studies are warranted to clarify the clinical utility of the ASA classification for perioperative risk stratification and postoperative care planning in pediatric patients undergoing dental treatment under general anesthesia.

## 5. Conclusions

In this retrospective observational study, higher ASA classification was significantly associated with postoperative intensive care unit admission in pediatric patients undergoing dental treatment under general anesthesia. As ASA classification inherently reflects preoperative systemic health status, this association should be interpreted within the context of structured perioperative risk stratification rather than as evidence of independent prognostic capability. Given the single-center retrospective design, further prospective multicenter studies are warranted to clarify the clinical relevance of this relationship.

## Figures and Tables

**Table 1:** Comparison of Children’s Intensive Care Needs, ASA Classification, and Associated Systemic Diseases by Gender.

		Gender		
		Male	Female	*p* *
Intensive Care Need Status	Yes	122 (20.9)	68 (16.3)	0.068
	None	463 (79.1)	350 (83.7)	
ASA Classification	I	398 (68)	307 (73.4)	0.325
	II	108 (18.5)	63 (15.1)	
	III	79 (13.5)	48 (11.4)	
Systemic Conditions in Children	None	391 (66.8) ^a^	303 (72.5) ^a^	<0.001
	Other	10 (1.7) ^a^	19 (4.5) ^b^	
	Allergy	22 (3.8) ^a^	18 (4.3) ^a^	
	Heart Problems	22 (3.8) ^a^	13 (3.1) ^a^	
	Autism	36 (6.2) ^a^	5 (1.2) ^b^	
	Cerebral Palsy	27 (4.6) ^a^	10 (2.4) ^a^	
	Epilepsy	28 (4.8) ^a^	17 (4.1) ^a^	
	Kidney Problems	5 (0.9) ^a^	4 (1) ^a^	
	Respiratory System Problems	8 (1.4) ^a^	1 (0.2) ^a^	
	Intracranial Mass	2 (0.3) ^a^	4 (1) ^a^	
	ADHD	7 (1.2) ^a^	1 (0.2) ^a^	
	Down Syndrome	14 (2.4) ^a^	3 (0.7) ^b^	
	Intellectual disability	13 (2.2) ^a^	20 (4.8) ^b^	

*: Chi-Square Test, ^a,b^: there is no difference between groups with the same letter.

**Table 2:** Comparison of Average Ages According to Children’s Gender.

	Male	Females	
	Mean ± SD	Mean ± SD	*p* *
Age	5.83 ± 2.98	5.72 ± 2.73	0.586

*: Mann–Whitney Test, SD: Standard deviation.

**Table 3:** Age Distribution According to Postoperative Intensive Care Unit (ICU) Admission and ASA Classification.

	Children’s Intensive Care Need Status		
	Yes	No	
	Mean ± SD	Mean ± SD	*p* *
ASA I	5.17 ± 2.00	-	-
ASA II	8.80 ± 4.80	5.70 ± 2.50	*p* < 0.001
ASA III	5.50 ± 0.70	7.70 ± 4.10	0.430
Overall	8.08 ± 4.32	5.24 ± 2.07	*p* < 0.001

*: Mann–Whitney Test, SD: Standard deviation.

**Table 4:** Association Between ASA Classification and Postoperative Intensive Care Requirement in Pediatric Patients.

		Intensive Care Need Status		
		Yes	No	*p* *
ASA Classification	I	0 (0) ^a^	705 (86.7) ^b^	*p* < 0.001
	II	66 (34.7) ^a^	105 (12.9) ^b^	
	III	124 (65.3) ^a^	3 (0.3) ^b^	

*: Chi-Square Test, ^a,b^: There is no difference between groups with the same letter.

**Table 5:** Multivariable Penalized Logistic Regression (Firth Method) Including ASA × Age Interaction for Postoperative Intensive Care Requirement.

		95% CI for EXP (B)		
	OR	Lower	Upper	*p*
Age	1.29	0.41	1.93	0.39
Gender	1.78	0.38	1.54	0.47
ASA	180.73	9.40	1922.49	*p* < 0.001
ASA × Age	1.02	0.82	1.70	0.89

OR: Odds ratio, CI: Confidence Interval.

## Data Availability

The datasets generated during the current study are available from the corresponding author upon reasonable request. The data are not publicly available due to privacy and ethical restrictions.
